# Case Report: Pallister-Hall syndrome diagnosed in adulthood during evaluation of recurrent hypoglycemia

**DOI:** 10.3389/fendo.2026.1925714

**Published:** 2026-09-25

**Authors:** Noha N. Mukhtar, Montaha Demyati, Allianah Benito, Ali S. Alzahrani

**Affiliations:** 1 Department of Medicine, King Faisal Specialist Hospital & Research Centre, Riyadh, Saudi Arabia; 2 Department of Medicine, Prince Mohammed Bin AbdulAziz Hospital, Almadinah, Saudi Arabia; 3 Molecular Endocrine Section, Research and Innovation, King Faisal Specialist Hospital & Research Centre, Riyadh, Saudi Arabia

**Keywords:** bifid epiglottis, *GLI3*, hypoglycemia, hypothalamic hamartoma, Pallister-Hall syndrome, polydactyly

## Abstract

**Introduction:**

Pallister-Hall syndrome (PHS) is a rare autosomal dominant disorder caused by pathogenic variants in *GLI3* gene. It is characterized by variable combinations of hypothalamic hamartoma, polydactyly, bifid epiglottis, renal and genitourinary abnormalities, imperforate anus, and pituitary dysfunction. Most cases are recognized during infancy or childhood; adult presentation is uncommon and hypoglycemia is not an established manifestation of PHS.

**Case description:**

A 26-year-old man with obesity and well-controlled hypertension was referred for evaluation of recurrent fasting and postprandial hypoglycemia associated with intermittent brief loss of consciousness. Physical examination revealed class III obesity, acanthosis nigricans, hand polydactyly, and a history of surgically removed extra toe in childhood. Family history was remarkable for polydactyly affecting the mother and multiple maternal relatives. Biochemical evaluation demonstrated recurrent hypoglycemia with inappropriately unsuppressed insulin and C-peptide concentrations, compatible with endogenous hyperinsulinemic hypoglycemia, during both prolonged fasting and mixed-meal testing. Abdominal magnetic resonance imaging showed no pancreatic lesion but an atrophic right kidney and hepatic steatosis. Whole-exome sequencing identified a heterozygous *GLI3* deletion with frameshift variant (NM_000168.6:c.2059del, p.(Glu687LysfsTer6)), confirming the diagnosis of PHS. Subsequent investigations demonstrated a hypothalamic hamartoma and partially bifid epiglottis, further confirming the diagnosis of PHS. The patient was referred for multidisciplinary management and genetic counseling.

**Conclusion:**

This case highlights an unusual late presentation of PHS with recurrent hypoglycemia, severe obesity, and episodic loss of consciousness and demonstrates the multi-systemic involvement in PHS. It emphasizes the importance of careful phenotypic assessment and the value of genetic testing in establishing a unifying diagnosis in complex presentations.

## Introduction

Pallister-Hall syndrome (PHS) is a rare autosomal dominant disorder first described in 1980 in patients with hypothalamic hamartoma (Initially referred to as hamartoblastoma), hypopituitarism, imperforate anus, and postaxial polydactyly (1, 2). This multisystemic developmental condition is caused by heterozygous pathogenic variants in *GLI3*, a zinc-finger transcription factor involved in Sonic hedgehog signaling (3–5).

The phenotypic spectrum of PHS is wide and includes hypothalamic hamartoma, mesoaxial or postaxial polydactyly, bifid epiglottis, imperforate anus, renal and genitourinary abnormalities, pulmonary segmentation anomalies, and pituitary dysfunction (3, 6). Initial reports described severe sporadic cases associated with major congenital anomalies, adrenal insufficiency, and early mortality (2, 4, 7). However, subsequent familial cases have described milder forms with excellent survival and marked intrafamilial variability (3, 8).

Hypothalamic hamartoma is a characteristic feature of PHS and may be associated with seizures, endocrine dysfunction, behavioral manifestations, obesity, and metabolic dysregulation (7, 9). The hypothalamus regulates appetite, autonomic tone, and glucose homeostasis through complex neuronal networks involving the arcuate nucleus, ventromedial hypothalamus, lateral hypothalamus, and paraventricular nucleus (10). Disruption of this complex network of neurons is likely the mechanism of many of the hypothalamic manifestations of PHS. Most cases of PHS are diagnosed during infancy and childhood, especially when family history of polydactyly or other congenital anomalies exists (3, 4). Late diagnosis of PHS is unusual but probably reflects unfamiliarity of the syndrome among physicians due to its rarity and the common occurrence of isolated polydactyly. In this report, we describe an unusual adult presentation of PHS in a 26-year-old man presenting primarily with recurrent hypoglycemia, obesity, and episodic loss of consciousness. During his evaluation for hypoglycemia, careful clinical assessment and whole-exome sequencing confirmed the diagnosis of PHS.

## Case description

A 26-year-old man without known diagnosis of PHS presented with a four-year history of well-controlled hypertension treated with amlodipine 5 mg daily when referred to our tertiary care center for evaluation of a one-year history of recurrent hypoglycemic attacks occurring two to four times per month. Episodes occurred during both fasting and postprandial states, with documented capillary blood glucose levels ranging from 2.7 to 3.3 mmol/L. Some episodes were associated with brief loss of consciousness lasting less than one minute, followed by spontaneous recovery regardless of food intake. Apart from mild dizziness, he reported no headache, focal neurologic symptoms, abnormal movements, palpitations, tremor, or other typical autonomic symptoms of hypoglycemia during these events. The patient did not undergo any weight-reduction surgery in the past and was not on any drugs that may explain hypoglycemia. The patient’s parents were non-consanguineous and his family history was notable for polydactyly in his mother, one sister, a maternal grandmother, and multiple maternal aunts and uncles, suggesting autosomal dominant inheritance.

Physical examination showed class III obesity (height 179 cm, weight 136 kg, body mass index 42.4 kg/m²), blood pressure 146/88 mmHg, mild acanthosis nigricans in the neck, right-hand polydactyly, and signs of surgically removed extra toe in childhood to allow him to wear comfortable shoes (**Figure 1**). Cardiovascular examination was unremarkable, and neurologic examination showed intact cranial nerve, motor, and sensory function.

**Figure 1:**
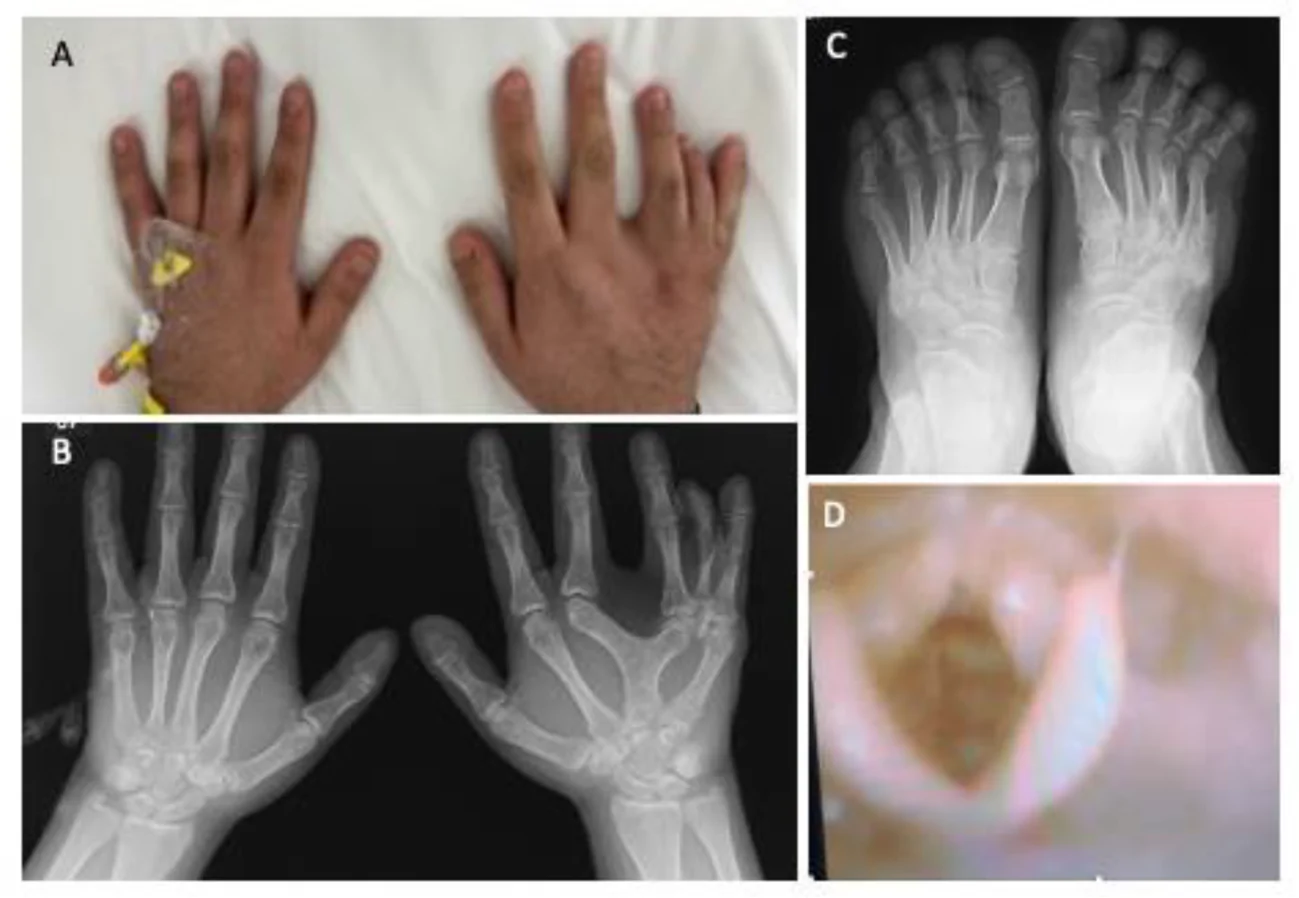
Clinical photograph and radiographs showing hand and foot polydactyly. **(A)** Clinical photograph of the hands. **(B)** Hand radiograph demonstrating fusion of the right third and fourth metacarpals with an insertional digit at the fourth metacarpal base, consistent with mesoaxial/central polydactyly. **(C)** Foot radiograph showing previous surgical resection of an extra right toe, consistent with postaxial/lateral polydactyly. **(D)** Partial bifid epiglottis on laryngoscopy.

Initial laboratory workup showed normal complete blood count and renal function (Creatinine 103 µmol/L, eGFR >60 mL/min/1.73 m²). Thyroid function and adrenal reserve were normal (thyroid-stimulating hormone 3.1 mU/L [reference range 0.2-4.2]; Free thyroxine 16.3 pmol/L [reference range 12-22]; morning cortisol 493 nmol/L). Other hormones were also within the normal range (Insulin-like growth factor 152 ng/mL [reference range for age and gender 130-295]; Testosterone 10.9 nmol/L [reference range 9.9-27]). Serum calcium was normal (2.35 mmol/L [reference range 2.1-2.6]), with high-normal parathyroid hormone (64.6 ng/L [reference range 15-65]) in the setting of vitamin D deficiency (20 nmol/L [reference range 30-120]). Random outpatient testing showed glucose 3.8 mmol/L with insulin and C-peptide in the higher range (Insulin 153 pmol/L, C-peptide 1.38 nmol/L), and glycated hemoglobin (HbA1c) was 4.9%. Prolactin level was 20.9 ug/L (4.1-18.4), adrenocorticotropic hormone was 42 ng/L (5-60), dehydroepiandrosterone sulfate was 11 umo/L (4.4-11.5). Luteinizing hormone, follicle-stimulating hormone and dynamic corticotropic and somatotropic testing were not performed.

To further evaluate recurrent hypoglycemia, the patient was admitted for a supervised 72-hour fasting test. A single critical hypoglycemic value was documented on the third day, 68 hours after starting fasting (blood glucose 2.4 mmol/L), associated only with mild dizziness. Glucose improved spontaneously without carbohydrate intake or intravenous dextrose injection, and he was able to continue fasting for another four hours until test completion without further decline in his blood sugar (**Table 1**). To investigate for reactive hypoglycemia, a mixed-meal test was performed the following day using a supplement containing 45 g of carbohydrate and this elicited mild dizziness and a drop in blood sugar to 2.8 mmol/L with concomitant insulin level of 163 pmol/L and C-peptide of 2.9 nmol/L (**Table 2**). Screening for insulin secretagogues was negative.

**Table 1:** Representative values from the 72-hour fasting test.

Date	Time	Plasma glucose (mmol/L)	Proinsulin (pmol/L; <18.8)	Insulin (pmol/L; 17-173)	C-peptide (nmol/L; 0.3-0.6)	BHB (mmol/L; 0.03-0.3)
7/12/2025	15:15	3.8	12.9	129	1.09	0.27
8/12/2025	17:25	3.8	11.8	86	0.90	1.44
9/12/2025	13:15	3.6	–	69	0.73	–
9/12/2025	15:55	2.4	6.3	73	0.72	–
9/12/2025	18:36	3.3	9.0	60	0.75	3.14

BHB, beta-hydroxybutyrate.

**Table 2:** Mixed-meal tolerance test.

Time	Plasma glucose (mmol/L)	Insulin (pmol/L; 17-173)	C-peptide (nmol/L; 0.3-0.6)
08:30 (baseline)	4.4	256	1.7
10:00	7.8	1314	6.1
10:30	10.4	2446	10.2
11:00	11.3	1752	12.3
11:30	9.1	3135	11.6
12:30	4.9	469	6.5
13:00	2.8	163	2.9
15:00	3.3	105	1.9

The test was performed using a supplement containing 45 g carbohydrate administered between 09:00 and 09:30.

At the critical fasting sample (glucose 2.4 mmol/L), insulin and C-peptide remained inappropriately unsuppressed, compatible with endogenous hyperinsulinism (14); β-hydroxybutyrate was not measured at this nadir, and the later value (3.14 mmol/L, obtained when glucose had risen to 3.3 mmol/L) cannot characterize ketogenesis during the critical sample. Insulin and C-peptide were also inappropriately high during postprandial hypoglycemia. For that reason, an abdominal magnetic resonance imaging (MRI) was performed to further screen for pancreatic tumors. MRI showed no pancreatic lesion, but revealed an atrophic right kidney with cortical scarring and diffuse moderate hepatic steatosis. Although negative magnetic resonance imaging does not exclude a small insulinoma, no additional localization study was performed due to the low index of suspicion.

The combination of recurrent hypoglycemia, obesity, acanthosis nigricans, hypertension, hepatic steatosis, polydactyly, renal abnormality, episodic loss of consciousness, and a strong maternal family history of polydactyly raised suspicion for an underlying genetic syndrome. Whole-exome sequencing identified a heterozygous *GLI3* deletion variant with frameshift and truncation [NM_000168.6:c.2059del, p.(Glu687LysfsTer6); rs2128709905; ClinVar Variation ID 1120244, classified pathogenic in ClinVar (18)], consistent with *GLI3*-related PHS. This was confirmed by Sanger sequencing (**Figure 2**).

**Figure 2:**
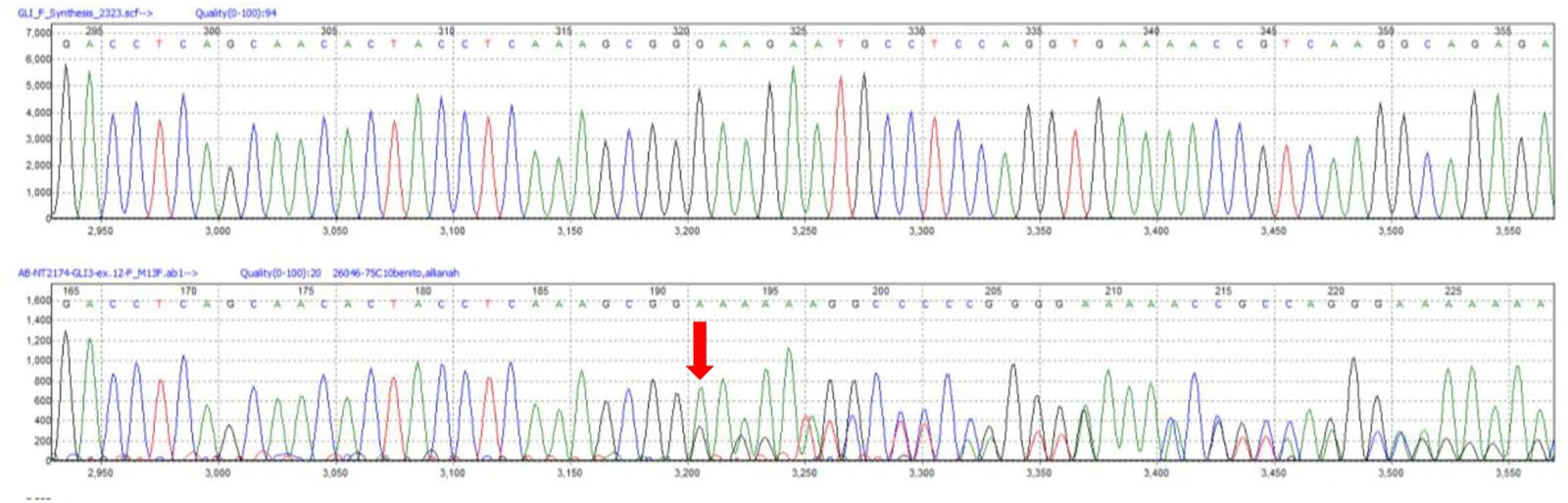
Part of a chromatogram of GLI3 exon 12 showing a heterozygous deletion with frameshift and truncation (NM_000168.6:c.2059del, p.Glu687LysfsTer6). The upper panel is reference sequence and the lower panel is the patient’s sequence.

## Management and follow-up

After identification of the *GLI3* variant, targeted screening for other manifestations of PHS was undertaken. Brain MRI demonstrated a well-circumscribed 12 × 12 × 7.5-mm hypothalamic lesion arising from the tuber cinereum with imaging features consistent with hypothalamic hamartoma (**Figure 3**). Flexible laryngoscopy demonstrated a partially bifid epiglottis (**Figure 1**). These findings strongly supported the diagnosis of PHS.

**Figure 3:**
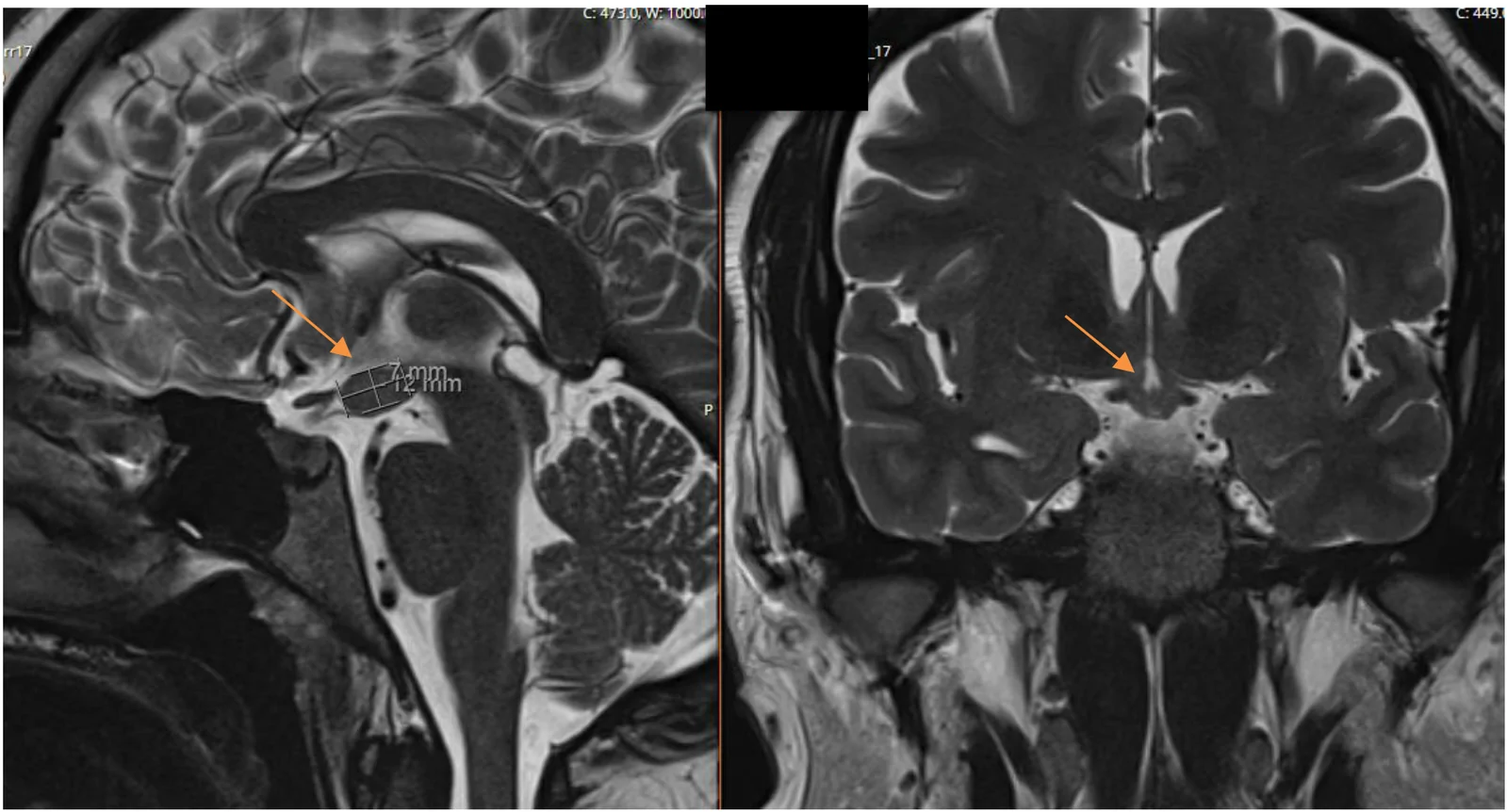
Sagittal and coronal T2-weighted brain MRI images show a well-defined hypothalamic lesion measuring approximately 12 x 12 x 7.5 mm, likely arising from the tuber cinereum and involving both mammillary bodies, with slight left-sided predominance. The lesion projects slightly anterior to the pituitary infundibulum. It is isointense to the adjacent cortex on T1- and T2-weighted sequences and shows no definite postcontrast enhancement. The pituitary stalk, not shown, is mildly thickened and deviated to the left.

Routine electroencephalography showed no epileptiform abnormalities. Antiseizure medication was not started; on subsequent 24-hour video-electroencephalographic monitoring, no habitual event, epileptiform abnormality, or hypoglycemia was captured, although the low event frequency limited the sensitivity of this study. He was referred to neurosurgery and medical genetics for further evaluation and genetic counseling.

The patient was advised on dietary modification to prevent hypoglycemia, including small frequent meals and avoiding simple carbohydrates. At six-month follow-up, he continued to report similar episodes with limited adherence to dietary measures; no additional pancreatic localization or neurosurgical intervention had been undertaken yet.

## Discussion

This case illustrates an unusual adult presentation of PHS diagnosed in the third decade of life after investigation of recurrent hypoglycemia and episodic loss of consciousness. Most patients with PHS are diagnosed during infancy or childhood because of congenital anomalies, polydactyly, endocrine abnormalities, or seizures (3, 4). Adult diagnosis has been reported but remains exceedingly rare (8).

The diagnosis in our patient was challenging because the biochemical and clinical findings initially appeared discordant. The patient had multiple features of insulin resistance and metabolic syndrome, including class III obesity, acanthosis nigricans, hypertension, and hepatic steatosis. These findings would usually argue against clinically significant endogenous hyperinsulinemic hypoglycemia. Nevertheless, fasting and mixed-meal testing showed recurrent hypoglycemia with declining but incompletely suppressed insulin and C-peptide concentrations.

Several features were atypical for a straightforward insulinoma, although none excluded it. First, β-hydroxybutyrate was not measured at the critical glucose nadir, so preserved ketogenesis cannot be inferred. Second, hypoglycemia improved spontaneously without carbohydrate intake or intravenous glucose injection. Third, abdominal MRI did not identify a pancreatic lesion. Finally, the coexistence of polydactyly, renal abnormality, family history of polydactyly, and episodic loss of consciousness suggested a syndromic pathology and this was confirmed by genetic testing. A small insulinoma, autoimmune hypoglycemia, and other causes of endogenous hyperinsulinism nevertheless remained incompletely excluded.

The identification of a *GLI3* frameshift variant provided a unifying diagnosis and prompted targeted evaluation that confirmed hypothalamic hamartoma and bifid epiglottis. The combination of hypothalamic hamartoma and mesoaxial polydactyly is highly characteristic of PHS, and molecular confirmation is established by identification of a heterozygous pathogenic or likely pathogenic *GLI3* variant (11), which was previously described (12). No peer-reviewed case reports with phenotypic description of another individual carrying this exact allele were identified; therefore, a direct allele-specific comparison of hypoglycemia or other endocrine manifestations was not possible.

GLI3 is a zinc-finger transcription factor located on chromosome 7p14.1 and is a key downstream mediator of the Sonic Hedgehog (SHH) signaling pathway, which plays a fundamental role in embryonic development and organogenesis (3, 5, 13). During normal development, GLI3 functions as both a transcriptional activator and repressor, regulating genes involved in limb formation, craniofacial development, hypothalamic differentiation, and formation of multiple midline structures (3, 6, 13). In the absence of SHH signaling, GLI3 is processed into a repressor form that suppresses target gene transcription, whereas activation of the SHH pathway promotes formation of the full-length activator form (13). Pathogenic truncating mutations in *GLI3* disrupt this tightly regulated balance and lead to abnormal embryologic development (3, 5). PHS is classically associated with truncating *GLI3* variants, particularly within the middle third of the gene, resulting in constitutive repressor activity and abnormal downstream SHH signaling (5, 15). This dysregulation explains the characteristic phenotypic features of PHS, including hypothalamic hamartoma, mesoaxial or postaxial polydactyly, bifid epiglottis, imperforate anus, pituitary abnormalities, and genitourinary malformations (3). In addition to PHS, *GLI3* mutations cause two additional distinct syndromes, Greig-Cephalopolysyndactyly syndrome (GCPS), and isolated polysyndactyly (IPD) (12, 13). GCPS is characterized by polysyndactyly, macrocephaly, and facial dysmorphisms including hypertelorism, broad nasal bridge, high forehead, and frontal bossing (16). The marked phenotypic variability observed in *GLI3*-associated disorders likely reflects differences in mutation location, residual GLI3 activity, modifier genes, and developmental timing of SHH pathway disruption (3, 6, 12, 15). Some studies showed that GCPS is predominantly attributed to variants affecting the N-terminal and C-terminal third of the GLI3 protein, whereas PHS is attributed to variants affecting the central third of GLI3 (6, 17). A more recent comprehensive assessment of *GLI3* variants confirmed the central third clustering of mutations for PHS but a more general involvement of all regions of the gene in case of GCPS and IPD (12).

Hypothalamic hamartomas are congenital, non-neoplastic malformations composed of disorganized neuronal and glial tissue (9). Depending on location and attachment pattern, they may cause gelastic or other seizure types, central precocious puberty, cognitive or behavioral symptoms, pituitary abnormalities, obesity, and other endocrine manifestations (7, 9). The brief episodes of loss of consciousness in our patient did not consistently fulfill Whipple’s triad and may represent hypothalamic seizure activity rather than purely neuroglycopenic events.

The mechanism of hypoglycemia in this case remains uncertain. Hypothalamic dysfunction may alter glucose sensing, autonomic counter-regulation, appetite control, and pancreatic islet signaling. Argente et al. have described the major role of AMP-activated kinase (AMPK) pathway in the paraventricular nucleus of the hypothalamus in glucose homeostasis, through integration of both central neuronal circuits and autonomic (sympathetic and parasympathetic) nervous system (10). A hypothalamic contribution to glucose regulation is therefore biologically conceivable but remains unproven, and no direct evidence of impaired autonomic counter-regulation was obtained in this patient. The hypothalamic hamartoma is therefore best regarded as a diagnostic feature of PHS rather than a demonstrated cause of the hypoglycemia. Although hypopituitarism can cause hypoglycemia, screening pituitary evaluation (morning cortisol, thyroid function, IGF-1, and testosterone) was unremarkable; however, LH, FSH, and dynamic corticotropic and somatotropic testing were not performed, so subtle pituitary dysfunction cannot be fully excluded, and long-term endocrine surveillance is recommended.

An additional notable feature in this case is mesoaxial or central polydactyly of the hand, a relatively characteristic feature of *GLI3*-related PHS (12). The previously resected extra toe was more consistent with postaxial polydactyly, which is less specific but frequently encountered in PHS and related *GLI3*-associated disorders which may be related to phenotypic variability of this mutation. The strong maternal family history of polydactyly further supports autosomal dominant inheritance with variable expression. However, family members were not available for direct examination and genetic testing.

PHS has historically been associated with severe congenital disease (2, 5, 7). Early reports included sporadic cases with major structural malformations and adrenal insufficiency leading to lethal outcomes (1, 2). However, subsequent experience has shown that PHS encompasses a wide clinical spectrum, with many patients having mild familial disease and long-term survival (3, 4, 8). Our patient highlights how mild or incomplete phenotypes may escape recognition until adulthood, particularly when polydactyly is treated surgically in childhood without appropriate evaluation for a possible underlying cause.

This case underscores the continuing value of meticulous bedside examination in endocrine practice. Recognition of polydactyly, family history, and renal and airway anomalies changed the diagnostic direction from isolated hypoglycemia to a syndromic diagnosis. It also demonstrates the value of genetic testing when clinical, biochemical, and radiologic findings do not fully explain the presentation.

## Conclusion

We describe an unusual late presentation of PHS in an adult man presenting with recurrent hypoglycemia of unclear pathogenesis, obesity, and episodic loss of consciousness. The diagnosis was established by integrating phenotypic findings, family history, biochemical evaluation, radiologic findings, and whole-exome sequencing. This case emphasizes that *GLI3*-related PHS may remain undiagnosed into adulthood and should be considered in patients with polydactyly, hypothalamic lesions, unexplained endocrine manifestations, and multi-systemic abnormalities.

## Data Availability

The relevant clinical and molecular data are included in the article. Individual-level genomic data are not publicly available to protect patient confidentiality but are available from the corresponding author on a reasonable request.
